# NMR-Based Metabolomic Analysis of Plasma in Patients with Adult Congenital Heart Disease and Associated Pulmonary Arterial Hypertension: A Pilot Study

**DOI:** 10.3390/metabo12090845

**Published:** 2022-09-08

**Authors:** Beizhu Xu, Caihua Huang, Caojin Zhang, Donghai Lin, Weifeng Wu

**Affiliations:** 1 Department of Cardiology, The First Affiliated Hospital of Guangxi Medical University, Nanning 530021, China; 2 Research and Communication Center of Exercise and Health, Xiamen University of Technology, Xiamen 361024, China; 3 Department of Cardiology, Guangdong Cardiovascular Institute, Guangdong Provincial People’s Hospital, Guangdong Academy of Medical Sciences, Guangzhou 510080, China; 4 Key Laboratory for Chemical Biology of Fujian Province, MOE Key Laboratory of Spectrochemical Analysis & Instrumentation, College of Chemistry and Chemical Engineering, Xiamen University, Xiamen 361005, China

**Keywords:** pulmonary arterial hypertension, congenital heart disease, ^1^H-NMR, metabolomics, biomarker, metabolite

## Abstract

Patients with unrepaired congenital heart disease (CHD) are prone to pulmonary arterial hypertension (PAH). The ovine pulmonary arterial smooth muscle cells exposed to increased pulmonary blood flow (PBF) exhibited hyperproliferation and metabolic alterations, but the metabolic disorders of patients with CHD and associated PAH (PAH-CHD) have not yet been fully understood. Adult CHD patients were prospectively included and divided into the PAH-CHD group (*n* = 24) and CHD group (*n* = 38), while healthy adults were included as healthy control (HC) group (*n* = 29). Plasma from each subject was prepared for nuclear magnetic resonance (NMR) detection. ^1^H-NMR spectra were acquired using 850 MHz NMR spectrometer. A total of 28 metabolites were identified from the NMR spectra and their relative concentrations were calculated and analyzed by multivariate and univariate statistical analyses and metabolic pathway analysis. Receiver operating characteristic (ROC) curve analysis and correlation analysis were performed to identify potential biomarkers and assess their roles in clinical assessment. Multivariate statistical analysis showed that the metabolic profile of PAH-CHD was altered relative to CHD or HC, while that of CHD was altered relative to HC. The identified characteristic metabolites were alanine, glucose, glycine, threonine and lactate, and the areas under the ROC curves (AUCs) were 0.769, 0.808, 0.711, 0.842 and 0.817, respectively. Multivariate ROC curve analysis showed AUCs ranging from 0.895 to 0.955 for the combination of these characteristic metabolites. The correlation analysis indicated that lactate and threonine were significantly correlated with mean pulmonary arterial pressure, pulmonary vascular resistance and N-terminal pro-B-type natriuretic peptide. The increased PBF could trigger global metabolic alterations in patients with CHD, which were more severe in patients with PAH-CHD. The characteristic metabolites have the potential to be biomarkers of PAH-CHD, which could be used for its noninvasive diagnosis, severity and prognosis assessment, thereby improving the management of PAH-CHD.

## 1. Introduction

Increased pulmonary blood flow (PBF) is prevalent in patients with congenital heart disease (CHD) that mainly involves atrial septal defect (ASD) with or without partial anomalous pulmonary venous connection (PAPVC), ventricular septal defect (VSD) and patent ductus arteriosus (PDA). A portion of them will develop pulmonary arterial hypertension (PAH) in several years if untreated or repaired late, accompanied by remodeled pulmonary vasculature with elevated mean pulmonary arterial pressure (mPAP, >20 mmHg) and pulmonary vascular resistance (PVR, ≥3 Wood Units) [1]. The left-to-right shunting through the congenital heart defect can increase PBF and raise shear stress to pulmonary vasculature, resulting in pulmonary artery remodeling and the occurrence of PAH associated with CHD (PAH-CHD) [2,3]. The shunting direction will be reversed if the PVR is high enough in the advanced stage of PAH-CHD when it is called Eisenmenger syndrome and considered an irreversible or incurable disease with poor prognosis [4,5]. For this reason, early diagnosis and treatment are crucial for the management of PAH-CHD in practice.

In an ovine model with CHD, left-to-right shunting promoted hyperproliferation of the pulmonary arterial smooth muscle cells (PASMCs) and increased workload on the right heart leading to pulmonary arterial medial hypertrophy and right heart remodeling [2]. The PASMCs from the ovine model exhibited metabolic alterations including a significant decrease in mitochondrial oxygen consumption, membrane potential and tricarboxylic acid (TCA) cycle function and a decrease in glycolytic lactate production [6]. Moreover, the serum metabolites of patients with PAH-CHD were significantly altered relative to their healthy counterparts, which involved the dysregulated metabolism of lipids, glucose, amino acids and phospholipids [7]. In other patterns of PAH (idiopathic and hereditary PAH), plasma concentrations of more than 50 metabolites were varied from health controls [8]. The lung tissues from PAH patients also showed metabolic alterations of glycolysis, TCA cycle, fatty acid metabolism and oxidation pathways [9]. In addition, the right ventricle from the murine PAH model demonstrated the dysregulated branched chain amino acid (BCAA) metabolism and reduced fatty acid oxidation that might contribute to the reduction in the TCA cycle reactions of right heart [10]. Therefore, alterations of metabolism and bioenergetics have been recognized as the universal hallmarks of PAH as revealed by metabolomic analyses [11]. However, more details about the metabolic alterations in patients with PAH-CHD need to be further depicted.

To date, invasive cardiac catheterization is still a routine procedure for patients with CHD to assess PVR, ventricular diastolic function, pressure gradients and shunt quantification prior to defect closure, which is also critical for the diagnosis, risk stratification and treatment of PAH [1]. Furthermore, the high value of N-terminal pro-B-type natriuretic peptide (NT-proBNP) is also observed in patients with PAH and right heart failure, which is one of the potent prognostic factors for these patients [12]. The discovery of novel biomarkers could be helpful for developing noninvasive alternative methods for the diagnosis, severity and prognosis assessment of PAH-CHD.

Biomarkers associated with metabolisms, which are often analyzed using serum or plasma samples from a peripheral vein, allow for the rapid and noninvasive diagnosis of multiple diseases such as coronary heart disease [13], diabetes mellitus [14], PAH [8] and tumors [15], and for monitoring their severity and treatment effect. Hence, we speculate that the left-to-right shunt in patients with CHD could induce metabolic alterations and trigger the shifting of concentrations of metabolites in plasma, and further change their metabolic profiles when PAH occurred, from which a panel of metabolites could be identified as the potential biomarkers for the noninvasive diagnosis, severity and prognosis assessment of PAH-CHD.

In the present study, we sought to exploit the metabolic alterations of patients with CHD and associated PAH by performing nuclear magnetic resonance (NMR)-based metabolomic analyses, and to identify a panel of metabolites as the potential biomarkers for metabolically distinguishing the PAH-CHD patients from CHD patients, and also for noninvasive diagnosis, severity and prognosis assessment. Our results may shed light on the clinical management of patients with PAH-CHD.

## 2. Subjects and Methods

### 2.1. Study Population

Adult CHD (ACHD) patients were prospectively included in this study, who were admitted to Guangdong Provincial People’s Hospital (Guangzhou, China) and underwent cardiac catheterization from 8 May to 31 December 2020, and the healthy adult volunteers were recruited during the same period as the healthy control (HC) group (*n* = 29). Here, CHD lesions refer to secundum ASD with or without PAPVC, VSD, PDA or coexisting two defects of them.

A total of 207 patients with unrepaired ACHD were consecutively included during the study period. We firstly excluded the patients complicated with systemic hypertension (*n* = 11), thyroid disorder or diabetes (*n* = 8), acquired heart disease (*n* = 4), cardiomyopathy (*n* = 1), severe anemia (*n* = 3), nephropathy or hepatopathy (*n* = 3), acute infection (*n* = 1), malignant tumor (*n* = 1), Down syndrome (*n* = 1), pregnancy (*n* = 1) and specific rheumatic diseases (*n* = 2), and also the patients without fasting blood left (*n* = 3). We then picked out the patients with large nonrestrictive defects, i.e., the defect diameter was larger than 20 mm in ASD, 10 mm in VSD or 6 mm in PDA, and excluded the remaining patients (*n* = 62 for ASD, *n* = 19 for VSD and *n* = 25 for PDA) [16]. The remaining 62 patients were subsequently divided into two groups: the PAH-CHD group (*n* = 24) and the CHD group (*n* = 38) according to the diagnostic criteria for PAH (mPAP > 20 mmHg, PVR ≥ 3 Wood Units and pulmonary artery wedge pressure ≤ 15 mmHg) recommended by the guideline [1]. 

This study was conducted in accordance with the Declaration of Helsinki and approved by the Medical Ethics Committee of Guangdong Provincial People’s Hospital (No. 2019-54-2, approval date: 15 November 2019). All of the patients and healthy volunteers gave informed consent to participate in this study and all of the patients had given written informed consent for the relevant diagnostic work-up and invasive interventional operations. 

### 2.2. Blood Samples Collection and Preparation

About 5 mL of elbow venous blood from each CHD patient or healthy person was drawn out after 8 h fasting, placed into a heparin anticoagulant tube and stored immediately in a refrigerator at 4 °C. Within half an hour, the blood sample was centrifuged at 3000 r/min (15 min, 4 °C). The upper plasma was extracted and put into the −80 °C refrigerator for later use.

Before the NMR experiment, the frozen plasma was thawed at 4 °C and then mixed by vortex. Then, 600 μL of plasma was used and centrifuged at 12,000 r/min (15 min, 4 °C). Thereafter, 400 μL of supernatant was accurately pipetted and transferred into 5-mm NMR tube followed by low-speed (1000 r/min) centrifugation (5 min, room temperature). A specialized inner tube was inserted into the 5-mm NMR tube. The solution in the inner tube contained 20% D_2_O for the locking magnetic field, 3-(trimethylsilyl) propionate-2,2,3,3-d4 (TSP) with a fixed concentration for providing NMR chemical shift reference (δ = 0.000) and quantifying metabolite levels and 50 mM phosphate buffer (pH 7.40).

### 2.3. NMR Measurements

All of the ^1^H-NMR experiments were performed at 25 °C on a Bruker Avance III 850 MHz spectrometer (Bruker BioSpin, Ettlingen, Germany) equipped with a TCI cryoprobe. One-dimensional (1D) ^1^H-NMR spectra were recorded using the Carr–Purcell–Meiboom–Gill (CPMG) pulse sequence [RD-90-(τ-180-τ)*n*-ACQ] with water suppression. RD was relaxation delay (4 s) and τ was spin echo delay (300 μs). A total of 32 transients were collected into 64K data points using a spectral width of 20 ppm with an acquisition time (ACQ) of 1.92 s. Two-dimensional (2D) ^1^H-^13^C heteronuclear single quantum coherence (HSQC) spectrum and ^1^H–^1^H total correlation spectroscopy (TOCSY) spectrum on a selected sample were also recorded to confirm the resonance assignments of metabolites identified from the 1D ^1^H-NMR spectrum.

### 2.4. NMR Data Processing

Free induction delay (FID) signals were processed by applying an exponential function with a line-broadening factor of 0.3 Hz prior to Fourier transformation. Then, the 1D ^1^H-NMR spectra were manually phased and carefully corrected for baseline distortion. Chemical shift correction was referenced to the methyl group of TSP at 0.0 ppm. 

The metabolites were identified using a combination of the typical 1D ^1^H-NMR spectra, 2D ^1^H-^13^C HSQC and 2D ^1^H-^1^H TOCSY spectra, the built-in library of Chenomx NMR Suite software (Version 8.3; Chenomx Inc., Edmonton, AB, Canada), the Human Metabolome Data Base (HMDB, http://www.hmdb.ca/ accessed on 26 November 2021) and relevant literatures [17,18,19]. 

Each NMR spectrum was binned by 0.001 ppm and integrated using the MestReNova software (Version 9.0; Mestrelab Research S.L., La Coruña, Spain). Thereafter, the spectral region of δ5.1–4.5 ppm (water resonance) was removed from the whole spectrum (δ9.5–0.0 ppm) to eliminate the distorted baseline from imperfect water saturation. Then, the integrals of metabolites were extracted from each 1D ^1^H-NMR spectrum by MATLAB (Version MATLAB2011b; MathWorks, Natick, MA, USA), which were normalized by the integral of TSP in each spectrum. The relative level of each metabolite was calculated based on both the relative integrals of singlet or nonoverlapped peaks of this metabolite and the number of protons contained in the corresponding hydrogen-containing group(s) of the metabolite, which was represented as mean ± standard deviation (SD) for each group of plasma samples.

### 2.5. Multivariate and Univariate Statistical Analyses

Multivariate statistical analysis was conducted with the SIMCA-P+ 14.1 software (Umetrics AB, Umea, Sweden). Pareto scaling was applied to increase the magnitudes of the low-abundance metabolites without significant amplification of noise. Unsupervised principal component analysis (PCA) was performed firstly to reveal the trends of metabolic separation, highlight outliers and show clusters among the samples. Supervised partial least-squares discriminant analysis (PLS-DA) was subsequently performed to optimize the metabolic separation between two groups of samples and extract the correlated variables related to sample belongings. The PLS-DA models of the two groups built on PLS regression were cross-validated to evaluate their robustness with response permutation tests (RPTs) of 200 cycles. Based on the validated PLS-DA models, significant metabolites were identified with the criterion of variable importance in projection (VIP) >1. 

The univariate statistical analysis was carried out with the SPSS 22.0 software (Chicago, IL, USA). Student’s *t*-test was performed to compare the relative concentrations of metabolites between two groups. Metabolites with the *p* value < 0.05 were identified to be differential metabolites. The metabolites belonging to both the differential metabolites and the significant metabolites were identified as characteristic metabolites.

### 2.6. Metabolic Pathway Analysis

Metabolic pathway analysis was performed to identify significantly disturbed metabolic pathways based on the relative concentrations of assigned metabolites using the module of Pathway Analysis provided by MetaboAnalyst 5.0 (https://www.metaboanalyst.ca accessed on 7 July 2022). Significantly altered metabolic pathways were identified with the two criteria of *p* value < 0.05 calculated by the metabolite set enrichment analysis and pathway impact value (PIV) > 0.2 computed by pathway topological analysis using the relative-betweenness centrality algorithm, which are well integrated in the module of Pathway Analysis. 

### 2.7. General Statistical Analysis

Statistical analysis was performed on SPSS 22.0 unless otherwise stated. Numerical variables were expressed as mean ± SD (if normally distributed) or median (interquartile range) (if not normally distributed), while the categorical variables were expressed as *n* (%). For quantitative comparison between the two groups, data were analyzed by Student’s *t*-test or Mann–Whitney U test. For the quantitative comparison among three groups, the data were analyzed by one-way ANOVA, followed by LSD post-hoc test. Chi-square test was used to compare the rates (%) between two or more groups. 

To explore potential biomarkers of PAH-CHD, we performed ROC curve analysis using the module of biomarker analysis on MetaboAnalyst 5.0 webserver (https://www.metaboanalyst.ca accessed on 7 July 2022). Univariate ROC curve analysis was employed to calculate the area under the curve (AUC) for each characteristic metabolite identified from the comparison of PAH-CHD vs. CHD, followed by multivariate ROC curve analysis that was used to calculate the AUCs for the combination of more than two characteristic metabolites by Monte Carlo cross validation (MCCV), using balanced sub-sampling. In each MCCV, two-thirds of the samples were used to screen out significant features that were then employed to build the classification model. The remaining one-third of the samples were employed to validate the classification model. We selected the PLS-DA method as the classification method and feature ranking method, and used the top five features (the characteristic metabolites previously identified) to generate the ROC curves. 

The correlation analysis was also conducted to explore the linear correlations of characteristic metabolites with some clinical parameters related to diagnosis and prognosis of PAH-CHD through calculating their Pearson correlation coefficients and *p* values with GraphPad Prism (version 8.0.2; GraphPad Software, San Diego, CA, USA). Correlations with *p* < 0.05 was considered statistically significant.

## 3. Results

### 3.1. Characterization of Subjects in Three Cohorts

A total of 91 subjects were included in this study and divided into three groups. The average age was not significantly different between PAH-CHD and CHD groups (*p* > 0.05), but greater than that of the HC group (*p* < 0.05). As shown in Table 1, it was female-predominant in both the PAH-CHD and CHD groups. Obviously, the body mass index (BMI) of the PAH-CHD group was lower than that of the CHD group or HC group (both *p* < 0.05). No statistical difference in BMI was observed between CHD and HC groups (*p* > 0.05). The pre-tricuspid defect (secundum ASD) was the main cardiac defect in both PAH-CHD and CHD groups (*p* > 0.05). Compared to the CHD group, the PAH-CHD group showed lower values of the mixed venous oxygen saturation (SvO_2_) (*p* < 0.05) and the arterial oxygen saturation (SaO_2_) (*p* < 0.001), but significantly higher values of the mPAP, PVR and right ventricular stroke work index (RVSWI) (all *p* < 0.001). No statistically significant difference in cardiac index (CI) was observed between PAH-CHD and CHD groups. Note that 4 out of 24 patients (16.7%) with PAH-CHD received targeted therapy before blood sampling.

### 3.2. Resonance Assignments of Metabolites

A total of 28 metabolites were identified based on typical 1D ^1^H-NMR spectrum (Figure S1) and confirmed by 2D ^1^H-^13^C HSQC and ^1^H–^1^H TOCSY spectra (Figures S2 and S3). The relative levels of the identified metabolites are shown in Table S1. The variation of relative plasma level of each metabolite is displayed together with its chemical shift and VIP value in Table 2.

### 3.3. Metabolic Shifting Induced by Increased Pulmonary Blood Flow 

**Figure 1:**
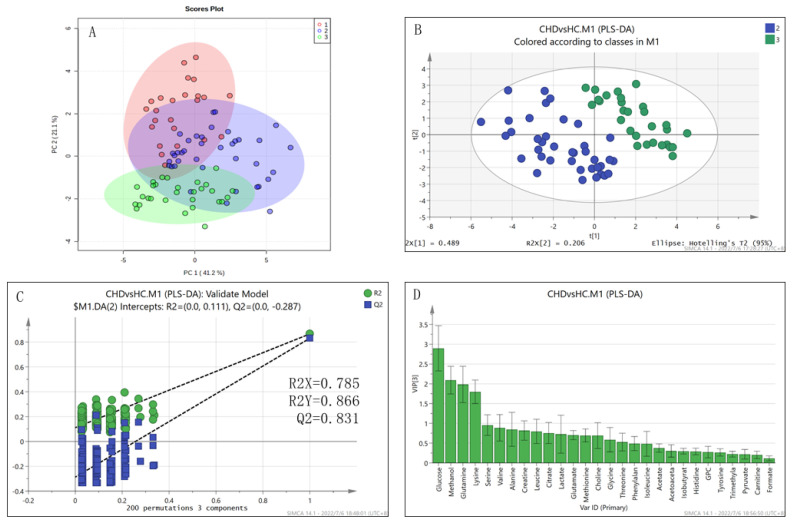
Multivariate statistical analysis of metabolite levels for the CHD and HC groups. (**A**) Unsupervised PCA scores plot of all samples. The PAH−CHD, CHD and HC groups of samples are marked in red, blue and green, respectively; (**B**) Supervised PLS−DA scores plot of CHD (blue) vs. HC (green); (**C**) Robustness of the PLS−DA model validated by RPTs with 200 cycles; (**D**) VIP values of metabolites calculated from the PLS−DA model.

All of the patients in the CHD group had suffered from congenital pulmonary over-circulation until they reached adulthood. The scores plot of the unsupervised PCA model shows metabolically distributing trends of 91 samples (Figure 1A), which were further verified by supervised PLS-DA when compared pairwise. 

The metabolic shifting between the CHD and HC group was confirmed by the robust PLS-DA model (Figure 1B) passing RPTs with 200 cycles (Figure 1C). The levels of 26 metabolites were significantly increased (all *p* < 0.01, Table 2) in CHD patients, in which 4 metabolites (glutamine, lysine, methanol, glucose) were identified to be characteristic metabolites (Figure 1D, Table 2). The identified significantly altered metabolic pathways included: D-glutamine and D-glutamate metabolism (PIV = 0.50, *p* = 2.770 × 10^−15^); alanine, aspartate and glutamate metabolism (PIV = 0.31, *p* *=* 1.366 × 10^−12^); phenylalanine, tyrosine and tryptophan biosynthesis (PIV = 1.00, *p* = 4.381 × 10^−10^); phenylalanine metabolism (PIV = 0.36, *p* = 4.3814 × 10^−10^); glycine, serine and threonine metabolism (PIV = 0.25, *p* = 3.503 × 10^−5^); histidine metabolism (PIV = 0.22, *p* = 1.651 × 10^−4^) and synthesis and degradation of ketone bodies (PIV = 0.60, *p* = 3.811 × 10^−4^), which were mainly involved in amino acid metabolism and fatty acid metabolism (Figure S4). Specifically, the accumulation of essential amino acids (leucine, isoleucine, valine, lysine, threonine, serine, phenylalanine) including three branched-chain amino acids (BCAAs) was observed in CHD patients; the changing plasma levels of pyruvate, citrate, glutamine, glutamate and histidine were indicative of the enhanced TCA cycle, pentose phosphate pathway (PPP) and purine metabolism; the impaired fatty acid metabolism was also observed in CHD patients, as supported by the increased levels of carnitine, acetoacetate, acetate and methanol. Moreover, the increased levels of one-carbon unit donors such as threonine, serine, glycine, histidine and methionine suggested promoted one-carbon metabolism. In addition, the upregulated levels of phenylalanine and tyrosine indicated enhanced tyrosine metabolism and more production of tyrosine-derived hormones which can affect metabolisms. 

**Figure 2:**
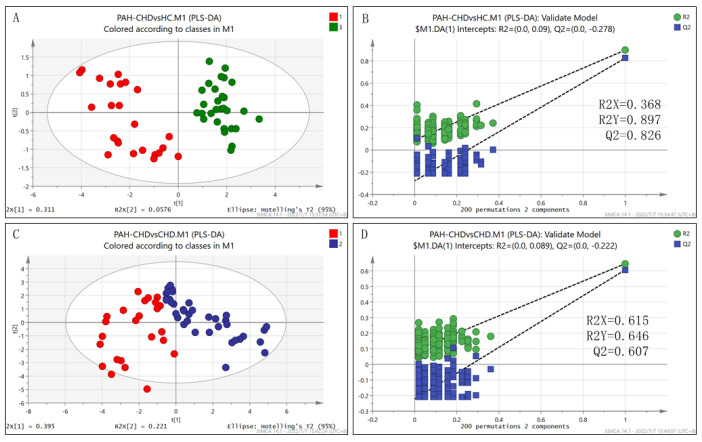
The scores plots of PLS−DA models and validations for pair-wise comparisons of the three groups. (**A**,**B**) PAH−CHD vs. HC; (**C**,**D**) PAH−CHD vs. CHD.

As PAH occurred, the patients with PAH-CHD showed a metabolic profile distinctly different from the health controls or patients with CHD only (Figure 2). Compared with health controls, 18 metabolites were increased in PAH-CHD patients, all of which were also increased in CHD patients except for lactate (Table 2). The increased lactate and other seven metabolites (glutamine, citrate, lysine, methanol, glucose, threonine and serine) were identified as the characteristic metabolites. The identified significantly altered metabolic pathways included: alanine, aspartate and glutamate metabolism (PIV = 0.31, *p* = 5.580 × 10^−10^); phenylalanine, tyrosine and tryptophan biosynthesis (PIV = 1.00, *p* = 7.730 × 10^−10^); phenylalanine metabolism (PIV = 0.36, *p* = 7.725 × 10^−10^); D-glutamine and D-glutamate metabolism (PIV = 0.50, *p* = 2.271 × 10^−8^); synthesis and degradation of ketone bodies (PIV = 0.60, *p* = 7.963 × 10^−7^); pyruvate metabolism (PIV = 0.27, *p* = 8.938 × 10^−7^); glycine, serine and threonine metabolism (PIV = 0.246, *p* = 7.644 × 10^−6^); and histidine metabolism (PIV = 0.22, *p* = 1.931 × 10^−5^) (Figure S5). The PAH-CHD patients presented more severe metabolic disorders compared with the CHD patients. Obviously, more pyruvate flowed to lactate rather than acetyl-CoA that would enter the TCA cycle for oxidative phosphorylation and energy generation, and the accumulation of citrate and promoted glutaminolysis were also observed, implying the disturbed oxidative phosphorylation and abnormal energy metabolism in patients with PAH-CHD.

### 3.4. The Differential Metabolic Pattern Distinguishing PAH-CHD from CHD

**Figure 3:**
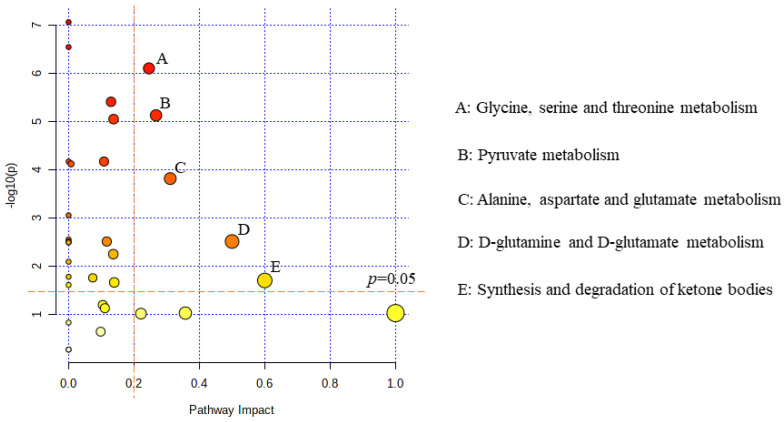
Metabolic pathway analysis for identifying significantly altered metabolic pathways in the PAH−CHD group relative to the CHD group. The vertical orange dashed line corresponds to the pathway impact value of 0.2 while the horizontal dashed line corresponds to the value of −log_10_(*p*) where *p* is equal to 0.05.

The PLS-DA model of PAH-CHD vs. CHD group was confirmed to be robust (Figure 2). Compared to the CHD group, the PAH-CHD group showed 10 decreased metabolites and 8 increased metabolites (all *p* < 0.05, Table 2). Alanine, glucose, glycine, threonine and lactate were identified as the characteristic metabolites between the two groups (Table 2) and the significantly altered metabolic pathways covered: glycine, serine and threonine metabolism (PIV = 0.25, *p* = 7.931 × 10^−7^); pyruvate metabolism (PIV = 0.27, *p* = 7.452 × 10^−6^); alanine, aspartate and glutamate metabolism (PIV = 0.31, *p* = 1.518 × 10^−4^); D-glutamine and d-glutamate metabolism (PIV = 0.50, *p* = 3.051 × 10^−3^); and synthesis and degradation of ketone bodies (PIV = 0.60, *p* = 1.955 × 10^−2^) (Figure 3). 

**Figure 4:**
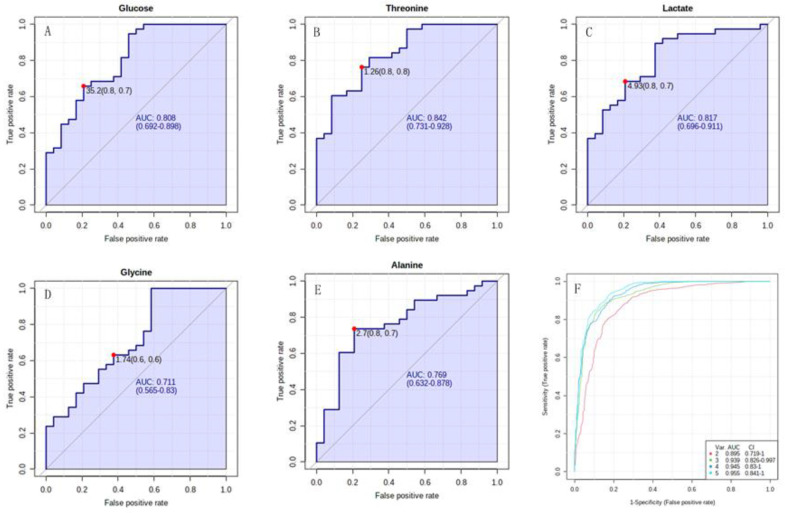
The ROC curve analyses of PAH−CHD vs. CHD. (**A**–**E**) Univariate ROC curves of five characteristic metabolites for distinguishing PAH−CHD from CHD; (**F**) Multivariate ROC curve of the combination of more than two characteristic metabolites. Each curve corresponded to each combination of different number of characteristic metabolites.

ROC curve analysis was performed to calculate the AUC for each characteristic metabolite and metabolites set to evaluate its diagnostic capability and potential to be a biomarker distinguishing PAH-CHD patients from the CHD cohort. The univariate ROC curve analysis showed that the AUCs for the characteristic metabolites were all above 0.700, while the multivariate ROC curve analysis displayed the enhanced results for the combination of two or more characteristic metabolites (Figure 4). The efficacy of classification reached the best result when AUC was 0.955 for using five characteristic metabolites simultaneously (Figure 4F). 

**Figure 5:**
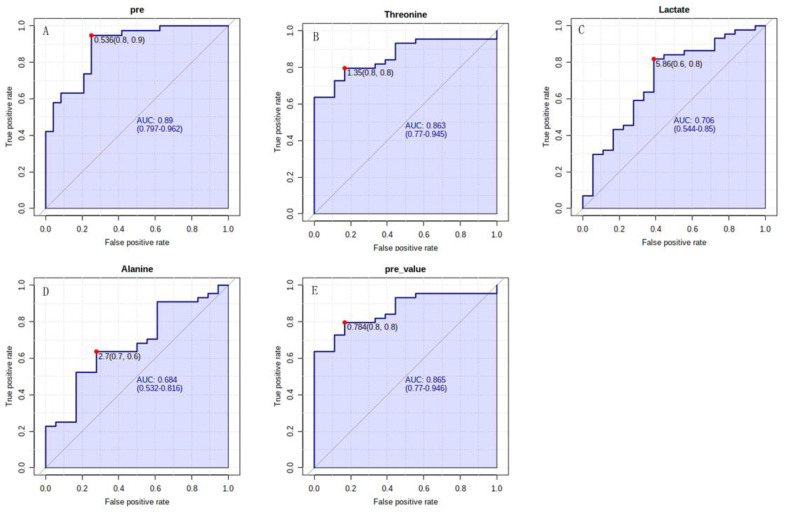
The ROC curve analyses of PAH−CHD vs. CHD. (**A**) The ROC curve generated by predictive values of probability from binary logistic regression model with lactate and threonine for diagnosis of PAH−CHD; (**B**–**D**) Univariate ROC curves of lactate, threonine and alanine for diagnosis of right heart dysfunction; (**E**) The ROC curve generated by predictive values of probability from binary logistic regression model with lactate, threonine and alanine for diagnosis of right heart dysfunction.

The diagnosis efficacy of PAH-CHD from CHD was obtained with a sensitivity of 0.8 and a specificity of 0.7 for lactate more than 4.93 relative level, and with sensitivity of 0.8 and specificity of 0.8 for threonine more than 1.26 relative level (Figure 4B,C). The diagnostic efficacy of PAH-CHD from CHD reached the better result with a sensitivity of 0.8 and a specificity of 0.9 for using both lactate and threonine (Figure 5A). Since NT-proBNP is the main index of risk stratification for predicting the annual death rate of patients with PAH and right heart dysfunction, we also assessed the diagnostic efficacy of PAH-CHD from CHD for the metabolites primarily responsible for determining the patients in medium to high risk with NT-proBNP more than 300 pg/mL. The obtained sensitivities and specificities were shown as follows: 0.8 and 0.8 for threonine with >1.35 relative level, 0.6 and 0.8 for lactate with >5.86 relative level, 0.7 and 0.6 for alanine with <2.7 relative level (Figure 5B–D). The sensitivity and specificity reached 0.8 and 0.8 when these metabolites were used for diagnosis simultaneously (Figure 5E).

### 3.5. The Characteristic Metabolites Were Correlated with Diagnosis and Prognosis of PAH-CHD

**Figure 6:**
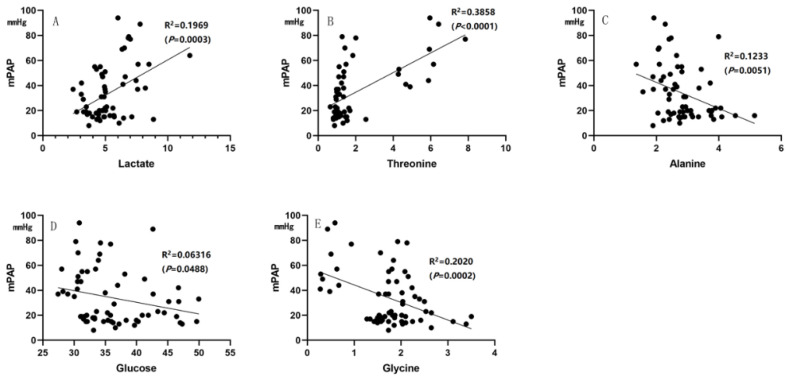
Correlation analysis. (**A**,**B**) Lactate and threonine were positively correlated with mPAP; (**C**–**E**) Alanine, glucose and glycine were negatively correlated with mPAP.

**Figure 7:**
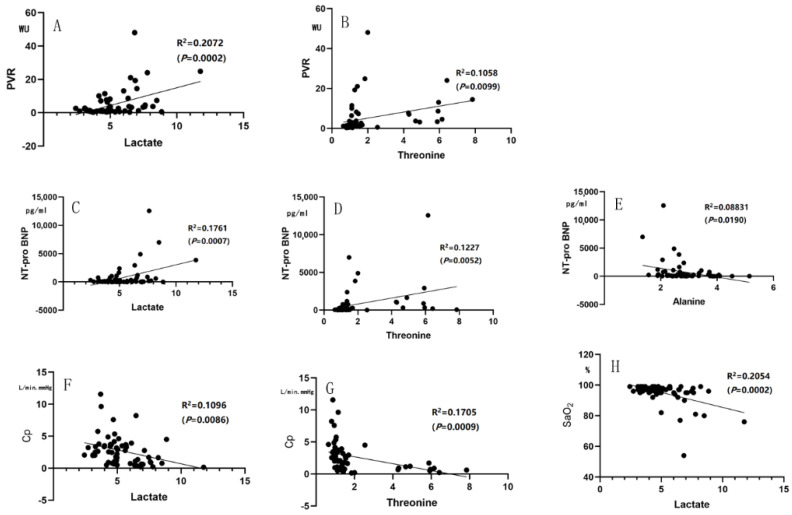
Correlation analysis. (**A**,**B**) Lactate and threonine were positively correlated with PVR; (**C**–**E**) Lactate and threonine were positively correlated with NT−proBNP, while alanine was negatively correlated with NT−proBNP; (**F**,**G**) Lactate and threonine were negatively correlated with Cp; (**H**) Lactate was negatively correlated with SaO_2_.

Some of the parameters from cardiac catheterization or clinical laboratories, such as mPAP, PVR, NT-proBNP, SaO_2_ and pulmonary arterial compliance (Cp), are associated with the diagnosis and prognosis (severity) of PAH in clinical assessment. In the correlation analysis, lactate and threonine were significantly correlated with mPAP, PVR, NT-proBNP and Cp (all *p* < 0.01), and lactate was significantly correlated with SaO_2_ (*p* < 0.01). Glucose and glycine were correlated with mPAP (both *p* < 0.05), while alanine was correlated with mPAP and NT-proBNP (both *p* < 0.05) (Figure 6 and Figure 7).

## 4. Discussion

The present study is an extension of metabolic profiling of PAH using NMR-based metabolomic techniques. The metabolic features of PAH-CHD were revealed by the metabolomic analysis of plasma from 24 adult patients with definite PAH and large nonrestrictive defects, whose pulmonary vasculature had been persistently exposed to increased PBF since their births. This represents the first study to our knowledge to illustrate the metabolic alterations of a specific PAH cohort including adult patients with left-to-right shunt using NMR-based metabolomic analysis. We also reported a group of metabolites that could be used as potential biomarkers for noninvasively distinguishing PAH-CHD from CHD, assessing its severity and predicting its outcome. 

Although we realize precisely that the increased PBF can cause proliferation and metabolic alterations of PASMCs in CHD models [2,6], the global metabolism of patients with CHD remains obscure. In our cohort, the CHD patients without PAH were observed with promoted metabolisms including energy metabolism and anabolism to meet the demands of pulmonary vascular cell proliferation and right ventricular overload. After the occurrence of PAH, these patients showed worsened metabolic disorders as reflected by obviously increased lactate and disturbed metabolic pathways. Thus, the metabolism of CHD patients is at the intermediate state between health and PAH-CHD. Most of the CHD patients will reverse pulmonary vascular remodeling after defect closure, while a substantial proportion of patients with PAH-CHD will eventually develop Eisenmenger syndrome or postoperative PAH with the worst prognosis [20,21].

The impaired glucose metabolism was observed in PAH-CHD patients with enhanced glycolysis and promoted lactate production, which were also previously reported in PAH models as the so-called Warburg effect found in cancer cells [22,23]. Nevertheless, our results suggest that the abnormal glycolysis of PAH-CHD may have some distinct features relative to other subtypes of PAH without cardiac shunting. Firstly, patients with large cardiac defect allowing more or less right-to-left shunting will decrease the systemic blood oxygen level and this condition will be worse when PAH occurs. Hence, the anaerobic glycolysis may more or less contribute to the elevated level of lactate. Secondly, some of the metabolic pathways usually consume the intermediate products of glycolysis in PAH-CHD, leading to the relative reduction in the end product of glycolysis despite the increased glucose uptake in the hyperproliferative pulmonary vascular cells [24,25]. The promoted PPP pathway and decreased downstream products of glycolysis were observed in PASMCs from the ovine model with increased PBF, which were also found in the lung tissues derived from patients with severe PAH [6,9]. To be specific, more 3-phosphoglycerate, a late-stage glycolytic intermediate, was much more likely to be transformed into serine in PAH-CHD, as reflected by both the downregulated level of 3-phosphoglycerate in the lung tissue and the disordered metabolic pathway including glycine, serine and threonine metabolism closely associated with the pathogenesis of PAH-CHD [9]. Therefore, less carbons derived from glucose are converted to acetyl-CoA entering the TCA cycle; thus, a decrease in oxidative phosphorylation of glucose exists in PAH-CHD patients.

Increased glutamate metabolism, which has been initially described in cancer cells proliferating rapidly, is also found in pulmonary arterial cells, lung tissue and right ventricle from PAH patients [26,27,28,29]. Glutamate is derived from glutamine by glutaminase and then converting to α-ketoglutarate (α-KG) by glutamate dehydrogenase [30]. The promoted glutaminolysis is closely associated with the inhibited TCA cycle in the conversion of citrate/isocitrate to α-KG which consumes oxygen and generates CO_2_, and maintains the continuous reactions of the TCA cycle by supplementing α-KG to the TCA cycle. On the one hand, the accumulating citrate transfers to cytoplasm and turns into acetyl-CoA that promotes fatty acid metabolism and supplies energy and lipid for cell growth [31]. On the other hand, promoted glutaminolysis can reduce the oxygen consumption and inhibit the generation of reactive oxygen species (ROS), thus contributing to survival of proliferative cells. The promoted glutaminolysis was also observed in both the CHD and PAH-CHD patients, in line with the hyperproliferative nature of pulmonary vascular cells and the hypertrophic nature of cardiomyocytes of the right ventricle, which were triggered by increased PBF. Besides, the upregulated levels of BCAAs in the plasma and tumor tissues are noticeable metabolic features in multiple neoplastic diseases, such as hepatocellular carcinoma [32], breast cancer [33] and myeloid leukemia [34], which promote cell proliferation by enhancing the energetic metabolism and biosynthesis of proteins and nucleotides in tumor tissues. Similar to cancer cells, the pulmonary vascular cells may accelerate their growth through inhibiting catabolism and raising the plasma levels of BCAAs in patients with PAH-CHD.

Furthermore, promoted one-carbon metabolism was previously observed in the pulmonary artery endothelial cells (PAECs) derived from PAH patients [35], which was further verified by our metabolomic data. The shifting of the glycine, serine and threonine metabolism in PAH-CHD patients reflects the increased one-carbon metabolism which is essential for biosynthesis processes including purine and thymidine synthesis and homocysteine re-methylation in rapidly proliferating cells [36], and is pivotal for REDOX balance during hypoxia [37].

Additionally, the increased levels of lysine, carnitine and acetoacetate indicate the imbalanced fatty acid metabolism, which was previously described in the hearts and lungs of PAH patients and also demonstrated by global metabolomics [9,38,39]. Carnitine, an essential cofactor for fatty acid metabolism, was increased in the PAH-CHD patients, which is in accordance with the results obtained from the other PAH-CHD cohort using ultra-performance liquid chromatography coupled high resolution mass spectroscopy (UPLC/MS) [7]. High levels of lysine and carnitine can promote fatty acid metabolism and enhance the energy supply by maintaining the integrity of mitochondrial respiratory chain to meet the energetic demand of rapid cell growth in PAH-CHD. In addition, the hypertrophic right heart mainly utilizes lactate and ketone bodies as fuels instead of fatty acids, contributing to the lipid accumulation and dysfunction of the cardiomyocytes [40,41]. Thus, the increased levels of lactate and ketone bodies indicate the disruption of fatty acid metabolism and inadequate contractile function of the right ventricle in PAH-CHD patients. This condition will become worse as the disease progresses.

NT-proBNP and BNP are considered as good markers for assessing right heart failure in PAH patients and their higher blood levels predict the poorer outcomes [42]. The potential metabolic biomarkers of lactate, threonine and alanine were well correlated with NT-proBNP, while lactate and threonine were also well correlated with mPAP and PVR, suggesting that the metabolic disorders are parallel with the disease severity, and these potential metabolic biomarkers may have diagnostic and prognostic values for the clinical management of patients with PAH-CHD. Expectedly, these biomarkers could be employed to noninvasively distinguish PAH-CHD from CHD patients alone or in combination. However, it may be more effective to use a panel of metabolites rather than a single metabolite, because these metabolites are the prominent parts of two distinct metabolic patterns with biochemical relations between each other. The metabolic pathway analysis further indicates that the metabolic alterations of PAH-CHD are inherently interrelated compared with CHD or HC. The distinct metabolic features of PAH-CHD patients mainly cover consumption of alanine, promotion in lactate production, enhanced one-carbon metabolism and abnormal energetic metabolism relative to CHD patients. Taken together, these characteristic metabolites could be used as potential biomarkers for the noninvasive screening of PAH, disease monitoring and prognosis assessing.

In clinical settings, the measurement of a series of plasma metabolites may be a new noninvasive alternative method to cardiac catheterization for those CHD patients with suspicious PAH. It is more efficient to make a diagnosis of PAH by detecting both lactate and threonine than one of them, as is to assess the risk of right ventricular dysfunction with NT-proBNP above the cutoff of 300 pg/mL, which is the critical index for risk stratification of patients with PAH [42].

A few limitations are associated with the present study. Aiming to explore the metabolic alterations of patients under long-term pulmonary over-circulation and associated PAH, we set the rigorous entry and exclusion criteria to reduce heterogeneity between patients in the same group, excluding most of the CHD patients from this study. Within an expected period of less than 8 months for performing blood sampling, we finally included only 24 PAH-CHD patients and 38 CHD patients without PAH in this study, resulting in an absence of validation cohort for the metabolic biomarker analysis. However, multivariate ROC curve analysis with MCCV using balanced sub-sampling could compensate for this imperfection to some extent. The use of an 850 MHz NMR spectrometer with a high magnetic field and cryoprobe provided a higher sensitivity for the NMR detection of aqueous metabolites. Therefore, our results are still reliable, albeit for the relatively small cohorts in our study. Besides, this study did not cover the metabolic enzymes involved in the impaired metabolic pathways of PAH-CHD and CHD, as well as metabolic alterations in the lung or heart tissues. Nevertheless, the metabolic pathway analysis based on the well-established methods and previous literature make it reliable to predict the metabolic alterations of lung and heart tissues by measuring the metabolites in peripheral venous blood. Our further study will expand the sample size and perform animal experiments to clarify the molecular mechanisms underlying the metabolic reprogramming triggered by left-to-right shunting.

## 5. Conclusions

Long-term increased PBF can trigger the remodeling of the pulmonary artery and right ventricle accompanied by organic dysfunctions and metabolic alterations. A portion of the patients with CHD will develop PAH-CHD in their adulthood, which can induce significant metabolic disorders. The metabolic alterations of PAH-CHD patients are indicative of impaired glucose and fatty acid metabolism, enhanced one-carbon metabolism, promoted glutaminolysis and inhibited TCA cycle relative to CHD patients without PAH. We identified five characteristic metabolites from the two differential metabolic patterns as the potential biomarkers of PAH-CHD, which could be used alone or in combination to distinguish PAH-CHD from CHD effectively. Our results may be beneficial to developing a noninvasive method to make a preliminary diagnosis of PAH-CHD for CHD patients with suspected PAH. In addition, the potential biomarkers identified from characteristic metabolites are significantly correlated with the crucial parameters of PAH such as mPAP, PVR and NT-proBNP, which are thus closely related to the diagnosis and prognosis of PAH-CHD. Therefore, the five metabolites including lactate, alanine, threonine, glucose and glycine, might have the potential for acting as the novel biomarkers for diagnosing PAH-CHD patients, with a promising future. Finally, our study addressing the metabolic alterations of a particular PAH subtype may advance our understanding of the metabolic impairments in PAH, from which some promising potential biomarkers could be identified to improve the management of patients with PAH-CHD.

## Figures and Tables

**Table 1:** Demographic and clinical characteristics of grouped subjects.

Group	PAH-CHD	CHD	HC
*n*	24	38	29
Gender (female), *n* (%)	15 (62.5)	28 (73.7)	16 (55.2)
Age, yrs	34.5 (27.0)	34.0 (17.0)	25.2 (7.2) *
BMI, kg/m^2^	19.4 ± 3.0	21.5 ± 3.2 *	21.1 ± 1.5 **
Pre-tricuspid defect, *n* (%)	13 (54.2)	25 (65.8)	NA
mPAP, mmHg	54 (28)	18 (7) ***	NA
SvO_2_, %	65.6 ± 9.9	71.8 ± 6.1 *	NA
SaO_2_, %	95.0 (13.0)	97.0 (3.0) ***	NA
PVR, Wood Units	7.55 (10.45)	0.95 (0.68) ***	NA
CI, L/min/m^2^	2.83 ± 0.96	2.98 ± 1.00	NA
RVSWI, g.m/m^2^	3.11 (1.69)	1.38 (0.91) ***	NA
Targeted therapy, *n* (%)	4 (16.7)	NA	NA

Notes: * denotes *p* < 0.05 (vs. PAH-CHD), ** denotes *p* < 0.01 (vs. PAH-CHD), *** denotes *p* < 0.001 (vs. PAH-CHD), NA means “not available”. Abbreviations: PAH-CHD, pulmonary arterial hypertension associated with congenital heart disease; CHD, congenital heart disease; HC, healthy control; BMI, body mass index; mPAP, mean pulmonary arterial pressure; SvO_2_, mixed venous oxygen saturation; SaO_2_, arterial oxygen saturation; PVR, pulmonary vascular resistance; CI, cardiac index; RVSWI, right ventricular stroke work index.

**Table 2:** The chemical shift and variation of each metabolite level among the three groups.

Chemical Shift (ppm)	Metabolites	CHD vs. HC			PAH-CHD vs. CHD			PAH-CHD vs. HC		
		VIP	Vary	*p*	VIP	Vary	*p*	VIP	Vary	*p*
0.93	Leucine		↑↑	0.000		↓↓	0.000		—	ns
0.98	Isoleucine		↑↑	0.000		—	ns		↑	0.019
1.02	Valine		↑↑	0.000		↓↓	0.006		↑↑	0.008
1.04	Isobutyrate		↑↑	0.000		—	ns		↑↑	0.000
1.45	Alanine		—	ns	1.10	↓↓	0.000		—	ns
1.90	Acetate		↑↑	0.000		↑↑	0.001		↑↑	0.000
2.32	Glutamate		↑↑	0.000		—	ns		↑↑	0.000
2.35	Pyruvate		↑↑	0.009		—	ns		—	ns
2.43	Glutamine	1.94	↑↑	0.000		↓↓	0.003	1.56	↑↑	0.000
2.50	Citrate		↑↑	0.000		↑↑	0.007	1.04	↑↑	0.000
2.61	Methionine		↑↑	0.000		—	ns		↑↑	0.000
2.90	Trimethylamine		↑↑	0.000		↑	0.037		↑↑	0.000
2.98	Lysine	1.77	↑↑	0.000		—	ns	1.74	↑↑	0.000
3.25	GPC		↑↑	0.005		↓	0.024		—	ns
3.26	Carnitine		↑↑	0.000		—	ns		↑↑	0.000
3.36	Methanol	2.04	↑↑	0.000		↓↓	0.004	1.79	↑↑	0.000
3.39	Glucose	2.80	↑	0.018	3.35	↓↓	0.000	1.49	—	ns
3.42	Acetoacetate		↑↑	0.000		↑	0.022		↑↑	0.000
3.53	Glycine		↑	0.011	1.15	↓↓	0.002		—	ns
3.56	Threonine		↑↑	0.002	2.19	↑↑	0.000	2.09	↑↑	0.000
3.91	Creatine		↑↑	0.000		—	ns		↑↑	0.000
3.95	Serine		↑↑	0.000		↑↑	0.003	1.47	↑↑	0.000
4.05	Choline		↑↑	0.001		↓↓	0.001		—	ns
4.09	Lactate	1.08	—	ns	2.02	↑↑	0.000	2.22	↑↑	0.000
6.87	Tyrosine		↑↑	0.000		—	ns		↑↑	0.000
7.30	Phenylalanine		↑↑	0.000		—	ns		↑↑	0.000
7.73	Histidine		↑↑	0.000		↓	0.019		—	ns
8.43	Formate		↑↑	0.000		↑↑	0.000		↑↑	0.000

Notes: the variation in metabolite level: ↑ and ↓ denotes *p* < 0.05, ↑↑ and ↓↓ denotes *p* < 0.01, ns means “not significant”; only listing the values of VIP > 1. Abbreviations: PAH-CHD, pulmonary arterial hypertension associated with congenital heart disease; CHD, congenital heart disease; HC, healthy control; GPC, Glycerylphosphorylcholine; VIP, variable importance in projection.

## Data Availability

Data are contained within the article or Supplementary Materials.

## Supplementary Materials

The following supporting information can be downloaded at: https://www.mdpi.com/article/10.3390/metabo12090845/s1. Figure S1: The typical 1D ^1^H spectra recorded on the three groups of plasma and resonance assignments of metabolites; Figure S2: 2D ^1^H-^13^C heteronuclear single quantum coherence (HSQC) spectrum and resonance assignments of metabolites; Figure S3: 2D ^1^H-^1^H total correlation spectroscopy (TOCSY) spectrum and resonance assignments of metabolites; Figure S4: Metabolic pathway analysis for identifying significantly altered metabolic pathways in CHD relative to HC: Figure S5: Metabolic pathway analysis for identifying significantly altered metabolic pathways in PAH-CHD relative to HC; Table S1: Relative levels of metabolites identified from 1D ^1^H-NMR spectra.
