# Supplementary material for: NMR-Based Metabolomic Analysis of Plasma in Patients with Adult Congenital Heart Disease and Associated Pulmonary Arterial Hypertension: A Pilot Study
Source: Metabolites. 2022 Sep 8;12(9):845. doi: 10.3390/metabo12090845 (PMC9504385; doi:10.3390/metabo12090845)
Supplement: Supplementary file 1 [file metabolites-12-00845-s001.zip › metabolites-1881641-supplementary.pdf]

NMR-based Metabolomic Analysis of Plasma in Patients with Adult Congenital Heart Disease and Associated Pulmonary Arterial Hypertension: A Pilot Study

Beizhu Xu, Caihua Huang, Caojin Zhang, Donghai Lin and Weifeng Wu

5 Supplementary Figures;

1 Supplementary Table.

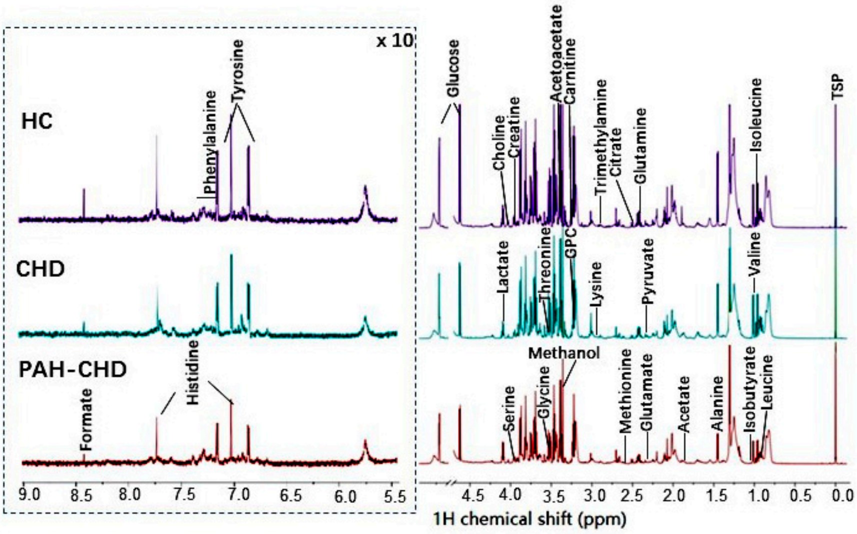

Figure S1. The typical 1D ^{1}H−NMR spectra recorded on the three group of plasma and resonance assignments of metabolites. Spectral region of δ5.1–4.5 ppm (water resonance) was removed and the region of δ9.0–5.3 ppm was amplified 10 times to make the peaks of low concentration metabolites easy to recognize. PAH−CHD, pulmonary arterial hypertension associated with congenital heart disease; CHD, congenital heart disease; HC, healthy control; GPC, glycerylphosphorylcholine.

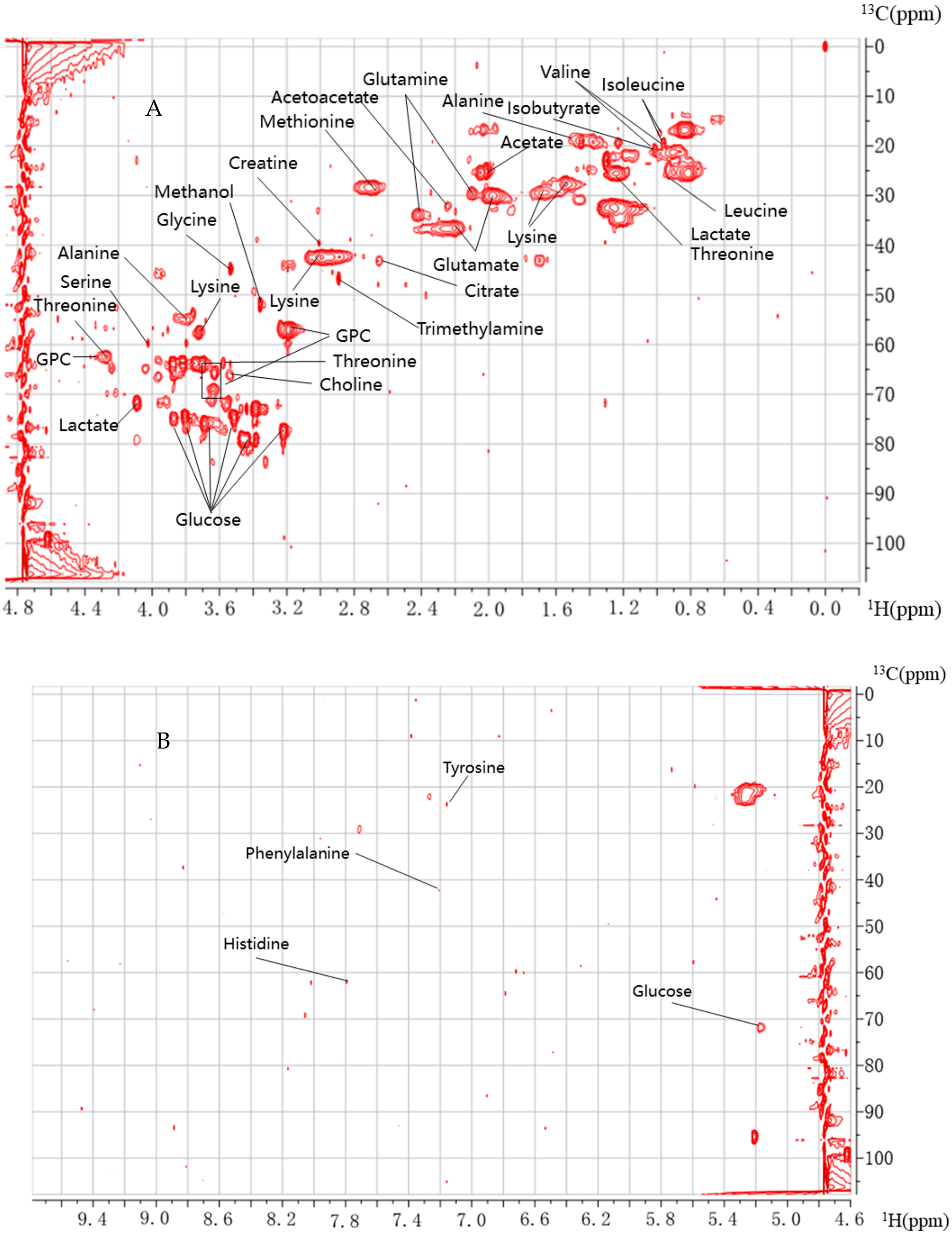

Figure S2. 2D ^{1}H-^{13}C heteronuclear single quantum coherence (HSQC) spectrum and resonance assignments of metabolites.

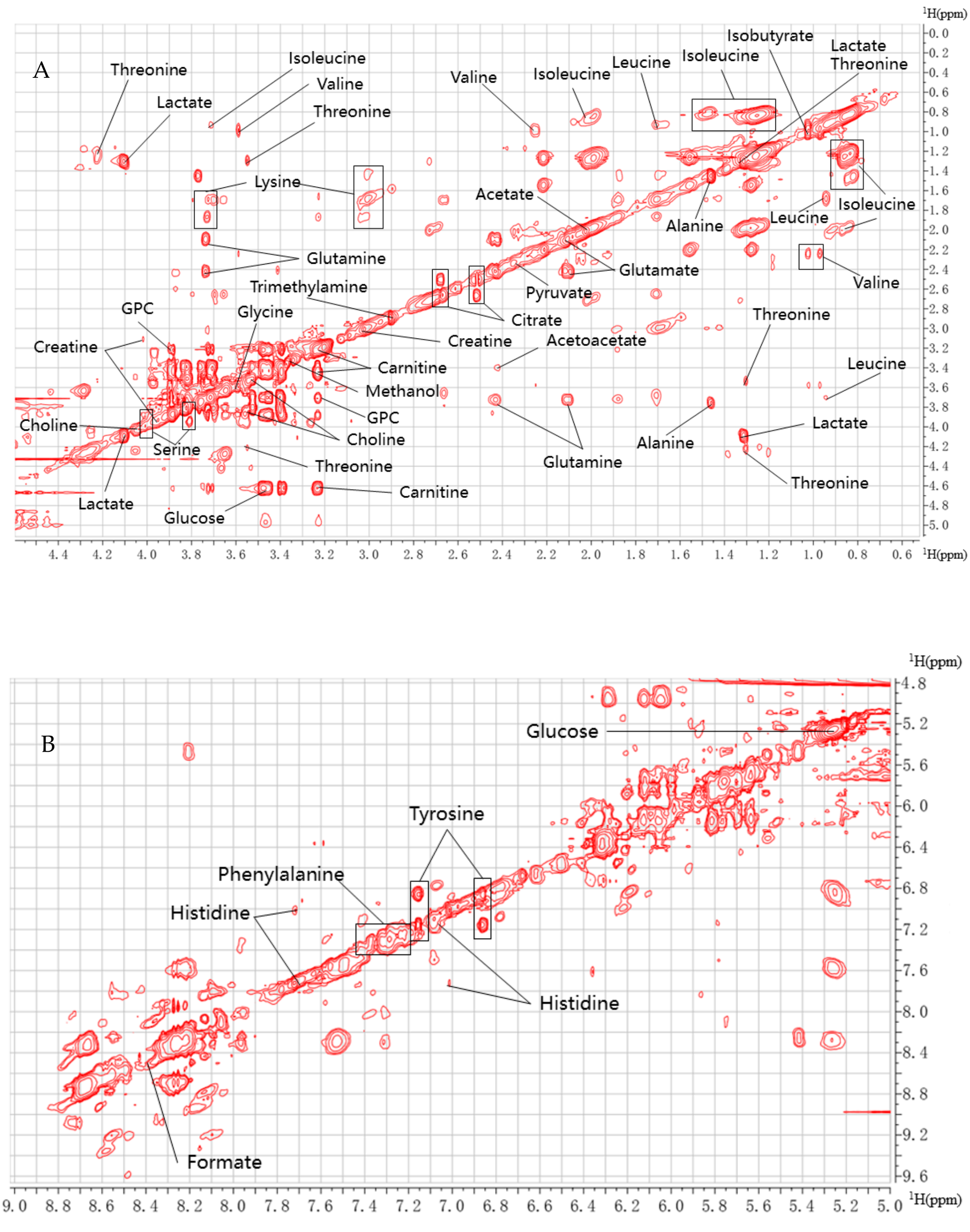

Figure S3. 2D ^{1}H-^{1}H total correlation spectroscopy (TOCSY) spectrum and resonance assignments of metabolites.

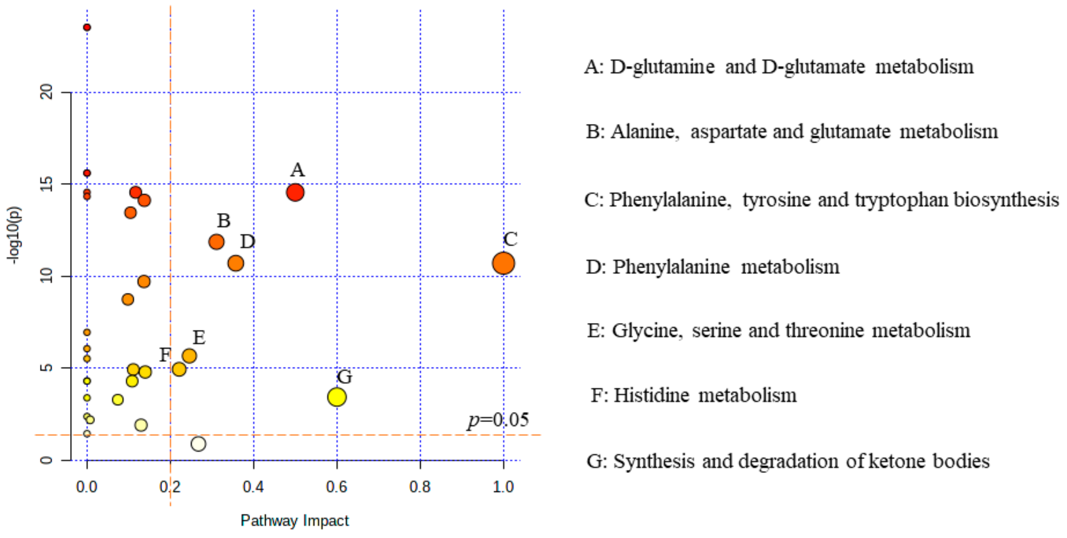

Figure S4. Metabolic pathway analysis for identifying significantly altered metabolic pathways in CHD relative to HC.

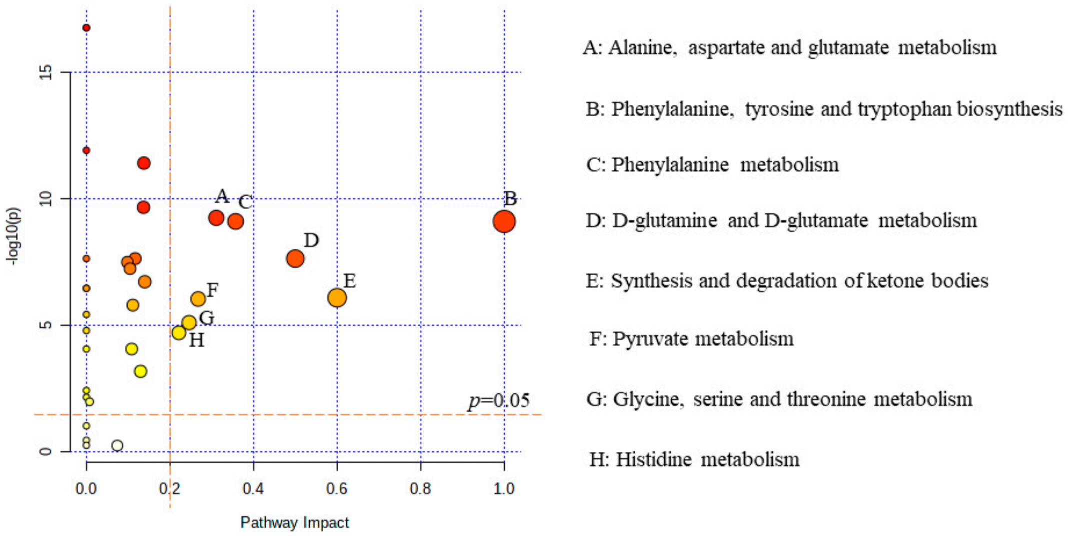

Figure S5. Metabolic pathway analysis for identifying significantly altered metabolic pathways in PAH-CHD relative to HC.

Table S1. Relative levels of metabolites identified from 1D ^{1}H-NMR spectra (mean ± SD)

**Metabolites**	**PAH-CHD**	**CHD**	**HC**
Leucine	1.258±0.280	1.554±0.371	1.246±0.202
Isoleucine	0.505±0.102	0.574±0.146	0.433±0.097
Valine	1.558±0.287	1.806±0.334	1.355±0.248
Isobutyrate	0.056±0.021	0.047±0.014	0.015±0.012
Alanine	2.444±0.570	3.071±0.723	2.722±0.563
Acetate	0.323±0.140	0.218±0.066	0.142±0.029
Glutamate	1.296±0.252	1.211±0.228	0.911±0.315
Pyruvate	0.260±0.039	0.280±0.071	0.238±0.084
Glutamine	4.298±0.758	4.881±0.700	3.271±0.487
Citrate	0.912±0.266	0.758±0.161	0.480±0.101
Methionine	0.499±0.093	0.547±0.083	0.354±0.061
Trimethylamine	0.047±0.011	0.041±0.009	0.022±0.007
Lysine	2.718±0.308	2.781±0.280	1.741±0.248
GPC	0.274±0.175	0.367±0.092	0.303±0.073
Carnitine	0.106±0.030	0.100±0.025	0.072±0.015
Methanol	1.989±0.420	2.293±0.483	0.863±0.504
Glucose	32.976±4.044	38.480±5.739	35.066±4.912
Acetoacetate	0.361±0.091	0.317±0.085	0.246±0.053
Glycine	1.285±0.735	2.070±0.653	1.705±0.242
Threonine	3.183±2.270	1.161±0.356	0.966±0.320
Creatine	0.576±0.149	0.557±0.143	0.323±0.069
Serine	3.031±0.364	2.731±0.394	2.202±0.328
Choline	0.539±0.390	0.792±0.467	0.484±0.150
Lactate	6.386±1.709	4.579±1.263	4.271±1.208
Tyrosine	0.254±0.059	0.239±0.048	0.192±0.038
Phenylalanine	0.162±0.074	0.137±0.050	0.053±0.023
Histidine	0.378±0.060	0.417±0.059	0.366±0.048
Formate	0.071±0.061	0.033±0.010	0.026±0.011

Abbreviations: PAH-CHD, pulmonary arterial hypertension associated with congenital heart disease; CHD, congenital heart disease; HC, healthy control; GPC, glycerylphosphorylcholine.
